# Industrial practitioner's perception on the application of exoskeleton system in automotive assembly industries: A Malaysian case study

**DOI:** 10.1016/j.heliyon.2024.e26183

**Published:** 2024-02-14

**Authors:** Woun Yoong Gan, Raja Ariffin Raja Ghazilla, Hwa Jen Yap, Suman Selvarajoo

**Affiliations:** Department of Mechanical Engineering, Faculty of Engineering, Centre for Sustainable and Smart Manufacturing (CSSM), University of Malaya, Kuala Lumpur, Malaysia

**Keywords:** Automotive manufacturing, Exoskeleton, Decision making, Assistive technologies, Wearable devices

## Abstract

The automotive industry is a key manufacturing industry for the Malaysian economy, where manual jobs and task are still common. Hence, Work-related Musculoskeletal Disorders (WMSD) is a common type of injury among workers. Exoskeleton system has gained global traction as a possible solution to reduce the risk of MSD among workers. Nonetheless, the application of exoskeleton in the automotive industry in Malaysia remains unknown. As such, this study attempts to provide insight into the industry's perception on the potential of exoskeleton application within the context of Malaysian automotive assembly sector. Therefore, a total of 52 management level respondents from various manufacturers participated in this study. It is found that, although the technology seems to be relatively new and disruptive, the respondents have a positive perception towards it with an acceptance rate of 86.5%. Cost of implementation exoskeleton technologies seems to be primary concern from the respondents, other concern such as maintenance cost and ease of application into existing application is also highlighted.

## Teaser

Practitioner Summary:The research intends to learn about the acceptance of exoskeleton in the automotive assembly industry in Malaysia. A survey was conducted to obtain the feedback from industry practitioners focusing on the management perspective. The findings suggest high acceptance of the application of exoskeleton in the industry with some concern on the cost.

## 1 Introduction

The automotive industry is one of the most important manufacturing sectors in Malaysia. Organisations and activities involved in the manufacturing of motor vehicles, such as passenger cars, light trucks, pickup trucks, vans, transport trucks, etc. are referred to as the automotive industry. The automotive industry is a potential industry that could contribute to the acceleration and evolution of Malaysia's economic and industrialisation processes, transforming the country into a developed nation. According to Statista, the Malaysian car sector has reached a saturation point. By 2019, there were 604 thousand motor vehicles in Malaysia, ranking third in the ASEAN automotive industry. Based on the data from MARii (Malaysia Automotive, Robotics and IoT Institute), Malaysia's automotive sector houses roughly 27 vehicle manufacturers, including passenger automobiles, commercial vehicles, and two-wheelers, producing approximately 500 thousand vehicles annually, making it the world's 23rd largest industry. Furthermore, it is reported that in the year 2018 the automotive industry provides opportunities of more than 755,000 jobs in manufacturing and aftersales [1].

Similar to other industries that require manual tasks, the workers in the automotive industry are also susceptible to Work-related Musculoskeletal Disorder (WMSD). WMSD is the most common problem faced by workers in an industrial environment due to repetitive and demanding work conditions. WMSD affects the upper limb extremities, lower back area, and lower limbs. Also, impairments of bodily structure such as muscles, tendons, joints, nerves, ligaments, bones, and the localised blood circulation system, induced or aggravated by tasks or the work environment can be characterised as WMSD [2].

Although the current industrial trend shifted towards automation and mechanisation, research [3] revealed that many workers are still subjected to physical demands like material handling, repetitive movements, and poor body posture. Although full automation could prevent work-related injuries while increasing efficiency, it is not always feasible. In certain manufacturing or warehouse environments that require a high level of flexibility, full automation would be prohibitively expensive to build or even impossible to implement. Thus, workers who are required to perform particular duties in an industrial environment are exposed to the risk of acquiring WMSD.

Studies [4] has found that Exoskeletons system to be a viable alternative solution towards WMSD reduction in manual tasks. The advantage of an exoskeleton is its flexibility in work/task adaption while maintaining the safe and efficient work through automation which could be a better option then robotic system. The exoskeleton is designed with the human anthropometric and biomechanics in mind to augment the human strength when worn [5]. Exo-system commonly comes in two type which is active or passive system. Active exoskeleton used drive system like motors, hydraulic or pneumatic systems while passive exoskeleton use elastic materials like springs or dampers to store and release energy. Some studies reported passive system is increasingly being adopted in automotive manufacturing industry in developed countries but were found to be less effective but could easily be integrated into the assembly lines because they are light, require no control, and have no standards bottleneck. Some of the passive exoskeletons were created to aid shop floor workers, while most designs are still prototypes that are yet to be built and put on the market [6].

However, exoskeleton technology is still considered new and fresh to the users in Malaysia and most of the industry practitioners are not familiar with the systems. Without any studies that focus on the demand and interest from automotive assembly industries, the application and the development of the suitable exoskeletons is limited and may not fit the working environment in automotive assembly industries.

## 2 Objective

This study aims to determine the acceptance of exoskeleton technology for the automotive assembly industry in Malaysia. Since there are many types of exoskeletons in the market, this survey aims to introduce and help to decide a suitable exoskeleton that could be incorporated into the industry in the future. Exoskeletons can help support workers with daily task operations by providing additional support. Besides the advancing technical developments of exoskeletons, the user's perspective needs to be considered too [7]. As such, the acceptance and usability of users are important factors in the application of new technology. Hence, the views of end-users and other stakeholders are important during the development [8]. This study collected responses from management, executive, and supervisory personnel who work in the automotive assembly industry on the characteristics of the exoskeleton and suitable applications that require an exoskeleton. The focus of this research is looking from a strategic operational perspective, which is why the respondents chosen were from the management level who will be key decision makers in the implementation of exoskeleton. This survey decided to choose management personnel rather than operator sampling as operators may have limitations in understanding the new technology, while from the supervisor level and above they have more understanding on the requirement of the overall production, tasks analysis, job design and proposal of new equipment that are suitable to be used in the production hence the data collected is much more suitable to meet the objective of the research.

## 3 Literature review

The automotive sector is one of the world's largest and most influential industries. The global automotive industries produce passenger automobiles such as sedans, SUVs, and commercial vehicles like trucks, buses, pick-up trucks, and other engine-based vehicles. The advancements in technology enable the automotive sector to manufacture electric cars, hybrid cars, auto-pilot vehicles, etc. However, engineers, technicians, operators and management teams are still required to operate and manage the industrial processes.

According to the MAA report [9], the average per annum production of vehicles in Malaysia from 2010 to 2020 was 513,714 units of passenger cars and 45,378 commercial vehicles. The highest production of passenger cars was recorded in the year 2015 with 563,883 units, while the lowest was 457,755 units in 2020. Meanwhile, the highest production of commercial vehicles of 59,999 units was recorded in the year 2012 and the lowest was 27,431 units in 2020.

The manufacturing procedures in the automotive sector begin with the selection of various materials [10]. The Research and Development (R&D) engineers are required to undertake extensive research on each component's range and design [11]. In most circumstances, the automotive sector outsources the production of those pieces to suppliers, where all the parts will be assembled into complete vehicles at the assembly factory [12]. Manufacturers can still choose to build some of the processes in-house. Press, body, power train, painting, and assembly shops are the most common key departments involved in the automotive assembly process, with each shop performing a particular role [13]. Meanwhile, there are also other sub-assembly stations for the assembly of various vehicle components including the logistics, Quality Control (QC), repair, and machining departments.

On the other hand, there are processes in the car assembly business that require workers to perform repetitive manual operations such as automation, semi-automation, and manual repetitive activities in the production of parts. The automation process successfully transformed the manual assembly process from man to machine, relieving the workers from the stress of heavy lifting. Hence, the tasks that cannot be automated and must be completed manually could lead to the risk of WMSD issues [14]. Approximately 34% of the annual lost time due to WMSD contributed to low worker productivity [15]. Workers who suffer from WMSD tend to slow down. WMSD is also responsible for 7% of the overall productivity loss [16]. Inefficient assembly method and lack of work satisfaction among the workers also results in a high level of absenteeism [17].

Workers in the automotive industry are exposed to occupational hazards such as WMSD due to severe force, non-neutral posture, repetition, and vibration while performing various tasks [18,19]. For instance, operators have to contend with awkward posture and repeated jobs in the automotive industry. WMSD in the automotive assembly line leads to serious ergonomic issues, due to hazardous duties such as tightening, picking up, material handling, and lifting [20]. Some other common processes that cause WMSD include overhead position tasks (increases the chances of shoulder/arm pain), tyre installation (increases the chance of wrist, shoulder, and arm pain), and auto seat installation (affects the muscles and joints in the shoulders, arms, back, and wrists). Meanwhile, lifting and transporting large components packaged in boxes at hip height, choosing and inserting goods from/into a pallet cage are just a few of the logistics duties that could lead to WMSD [21].

Posture and work environment are the causal reasons for WMSD. Hence, numerous possible options can be apprehended to improve the working environment, such as redesigning the workspace and improving working posture by providing workers with exoskeletons to complete daily activities. The exoskeleton is a wearable, external mechanical structure that allows users to clothe and support their body movement in the same way that a power suit does [22]. The early concept of an exoskeleton was majorly sketched as concept research and was never built or tested [23]. Yagn's running aid, patented in 1890, was the first device that resembled an exoskeleton. Then, General Electric began developing the prototype Hardiman exoskeleton in 1965, with the support from the U.S. Department of Defense, intending to provide users with superhuman strength. Applications and innovations were also discovered in the military sector to help soldiers improve their abilities. The medical exoskeleton was created to assist disabled or handicapped people with their rehabilitation and daily activities.

In the industrial sector, exoskeletons are used to support and assist workers in daily duties. There are two types of exoskeletons namely active and passive. The active exoskeleton has one or more actuators to help actuate human joints and increase the human's power. Electric motors, hydraulic actuators, pneumatic muscles, and other types of actuators can be used in the design of an active exoskeleton [24]. Whereas, a passive system does not use any kind of actuator, instead relies on materials, springs, or dampers to store the energy generated by human activities that can be used to support a posture or motion when needed. For instance, when a person bends forward, a passive exoskeleton may store energy which can be used to help the person maintain that position or upright the body while carrying an object.

Exoskeletons can be further classified based on the bodily parts they support, where lower-body exoskeletons support lower limbs, upper body exoskeletons support upper extremities, and full-body exoskeletons support both upper and lower extremities. Researchers have also designed and produced additional single-joint exoskeletons. The lower body exoskeletons are the most successful product utilised as rehabilitation tools to improve the quality of life for the crippled. Since the interest in industrial applications is growing rapidly, industries are ready to consider these exoskeletons for commercial development [25].

According to the literature, exoskeletons can improve cardiovascular health, energy consumption, body composition, gait characteristics, degree of physical activity, and quality of life [26]. Exoskeletons can be utilised as a rehabilitation tool to regain locomotion and to increase the level of physical activities years after an injury [27]. Exoskeletons may also aid in fighting against obesity syndrome following a spinal cord injury by reducing sitting time, increasing physical activity, and improving body composition metrics. According to the World Health Organization, muscular movement due to muscle activities that increases energy expenditure is referred to as physical activity. Exoskeletons allow passive lower-extremity movement without the use of muscles [28]. Powered exoskeletons would include straps to aid in maintaining a static and dynamic posture while standing and walking [29]. According to Pauline [30], an exoskeleton can reduce the physical stress on workers while simultaneously allowing them to maintain complete control over the technical gestures.

Some of the issues of exoskeletons include better power sources [31] and control algorithms [32] for quieter and smoother exoskeleton operation. The Direct Current (DC) batteries are most commonly used for actuators. The overall weight of the exoskeleton poses another problem making it less portable, apart from the cost of the exoskeleton [33]. Some exoskeleton designs necessitate the use of support crutches, providing the wearer with a sense of dependency. A majority of the exoskeleton prototype designs can affect or alter the biomechanics of a normal human stride [34]. Moreover, the sound of the exoskeletons could also make the users feel uncomfortable. Another constraint involves the information interchange between the wearer and the exoskeleton system, where the user's movement cannot be accurately and rapidly executed by the exoskeleton sensor. The other characteristics of an exoskeleton include the actuator's capacity to move [35] and the long-term power supply required to power up the exoskeleton. The regulatory organisations will only approve the application of exoskeleton at workplace after considering its safety [36]. According to literature, the precise alignment of an exoskeleton with the wearer's anatomical joints also remains a challenge [37].

## 4 Methodology

As exoskeleton technology is quite new to most industries in Malaysia, this study collected the perspectives of employees from different fields of the automotive industry who are aware of the technology.

The respondents who participated in this study were from the Malaysian automotive assembly industry comprising supervisors, engineers, executives, and management from passenger car, bus, and truck assembly sectors. The respondents from this survey were the management and executive working in the production line among the passenger cars, bus, and truck assembly industry. The respondents were combination of multinational and local assembly. The information of the respondents is stated in Table 1 below. The survey was done based on convenient sampling which involve management level executive which have decision making influence on the production system. A total of 52 respondents completed the survey from 18 among total 34 automotive assembly manufacturing in Malaysia. With 53 % coverage of organization in Malaysia, it is enough sufficient to provide exploratory insight to the problem. To avoid issues due to missing data, more than 70 questionnaires were distributed. Data were collected through online distribution which follow up via email. To provide some basic understanding of exoskeleton towards the survey, there are some basic introductions of exoskeleton technology before the respondent answer the survey and there's no specific type of exoskeleton focused on this study but more to exoskeleton technology in general.

**Table 1:** Basic information of automotive assembly industry.

Types of manufacturing	Number of manufacturers in Malaysia	Type of company		Brand	Number of staff	Number of respondents in this research
		MNC	Local/national			
Passenger car assembly industry	10	5	5	a	above 1000	31
				b	75–200	
				c	above 1000	
				d	above 1000	
				e	above 1000	
				f	above 1000	
				g	above 1000	
				h	above 1000	
				i	200–500	
				j	75–200	
Truck assembly	11	4	7	a	200–500	9
				b	75–200	
				c	0–75	
				d	above 1000	
				e	0–75	
				f	75–200	
				g	200–500	
				h	0–75	
				i	0–75	
				j	0–75	
				k	0–75	
Bus assembly	13	1	12	a	75–200	12
				b	75–200	
				c	0–75	
				d	500–1000	
				e	0–75	
				f	0–75	
				g	75–200	
				h	75–200	
				i	75–200	
				j	0–75	
				k	0–75	
				l	0–75	
				m	0–75	

The questionnaire for this study consists of three sections using Likert-scale method of a scale 1 to 5. The questionnaire also includes an open-ended section. The Likert-scale method is advantageous for respondents from production management as it is easier to understand based on the given options, so they can feedback in shorter time, also Likert-scale method is easier to quantify the results. Prior to commencing the survey, the questionnaire was pre-tested to ascertain its reliability and validity. Based on the results from the pre-test, some changes were made to avoid confusion among the respondents.

## 5 Results

### 5.1 Sociodemographic characteristics

The questionnaires were distributed to more than 70 employees who were working in the automotive industry. Of that 52 responses were collected which were mostly males, 48 (92.3%). The age range of the respondents was between 18 and 55, with a majority aged between 25 and 34 (52%). As for their designation, 8 respondents (15.4%) were supervisors, 28 (53.8%) were engineers, and 16 (30.8%) were at the management level. Meanwhile, 31 (59.6%) of respondents worked in the passenger car assembly industry, 9 (17.3%) in the truck assembly industry, and 12 (23.1%) in the bus assembly process. In terms of working experience, 18 respondents (34.6%) have been working in the industry for 1–5 years, followed by 13 (25%) respondents who have been in the industry for 6–10 years. According to 44 respondents (84.6%), their companies are not utilising the exoskeleton technology, while 2 (3.8%) mentioned that their companies are currently implementing the technology. The remaining stated that it is still under planning. Table 2 states the information of the respondents.

**Table 2:** Basic information of respondents.

Respondents		
	Frequency	Percentage (%)
Gender - Male - Female	48 4	92.3 7.7
Age − 18-24 − 25-34 − 35-50 − 51 above	8 27 15 2	15.4 51.9 28.8 3.8
Position - Supervisor - Engineer - Management	8 28 16	15.4 53.8 30.8
Industry - Passenger car assembly - Truck assembly - Bus assembly	31 9 12	59.6 17.3 23.1
Experience - Less than 1 year − 1–5 years − 6 to 10 years − 11 to 15 years - More than 15 years	9 18 13 4 8	17.3 34.6 25 7.7 15.4
Application of exoskeleton in industry - Yes - No - Planning	2 44 6	3.8 84.6 11.5

### 5.2 Benefits of exoskeleton technology

The respondents were asked regarding the benefits exoskeleton could offer the users and the industry. According to 34 respondents (65.4%), an exoskeleton could reduce the operation time lost due to injuries. While 29 respondents (55.8%) claimed that the exoskeleton could reduce the human energy expenditure requirement. Meanwhile, 25 respondents (48.1%) indicated that the exoskeleton could increase the maximum lifting load of workers. The other characteristics of exoskeletons that were rated important by the respondents included increasing work productivity (57.7%), reduction in WMSD cases (46.2%), increase in work quality (59.6%), reduction in muscle fatigue (63.5%), reduction in overall costs due to injuries (40.4%), and reduction in task duration (48.1%). Table 3 summarises the overall results.

**Table 3:** Benefit of exoskeleton.

Benefit of Exoskeleton	Not important at all		Not very important		Neither important nor unimportant		Very important		Extremely important	
	Freq.	%	Freq.	%	Freq.	%	Freq.	%	Freq.	%
1. Reduction in time lost due to injuries	0	0	1	1.9	6	11.5	34	65.4	11	21.2
2. Reduction in human energy expenditure requirement	1	1.9	1	1.9	7	13.5	29	55.8	14	26.9
3. Increase in maximum lifting load	1	1.9	1	1.9	7	13.5	18	34.6	25	48.1
4. Increase in work productivity	0	0	1	1.9	4	7.7	30	57.7	17	32.7
5. Reduction in WMSD cases	0	0	0	0	10	19.2	24	46.2	18	34.6
6. Increase in work quality	0	0	2	3.8	4	7.7	31	59.6	15	28.8
7. Reduction in muscle fatigue	0	0	0	0	4	7.7	33	63.5	15	28.8
8. Reduction in overall costs due to injuries	1	1.9	1	1.9	11	21.2	21	40.4	18	34.6
9. Reduction in task duration	1	1.9	0	0	8	15.4	25	48.1	18	34.6

### 5.3 Limitations of exoskeleton

More than half of the respondents (n = 28, 53.8%) claimed that they were concerned about the difficulties in maintaining and troubleshooting the exoskeleton. While 31 (59.6%) of them stated that the high cost of implementation could be a limiting factor. 27 respondents (51.9%) were very concerned about the possible limitation of movement of the exoskeleton. The overall results are tabulated in Table 4.

**Table 4:** Limitation of exoskeleton.

Limitation of exoskeleton	Not concerned at all		Not very concerned		Neither concerned nor not concerned		Very concerned		Extremely concerned	
	Freq.	%	Freq.	%	Freq.	%	Freq.	%	Freq.	%
1. Difficult to maintain and troubleshoot	2	3.8	2	3.8	9	17.3	28	53.8	11	21.2
2. Possible limitation in movement due to design	2	3.8	0	0	8	15.4	27	51.9	15	28.8
3. Lack of flexibility movement during accident	2	3.8	1	1.9	6	11.5	22	42.3	21	40.4
4. High cost of implementation and maintenance	1	1.9	2	3.8	6	11.5	12	23.1	31	59.6
5. Lack of size adjustability to suit different body figure	1	1.9	2	3.8	11	21.2	26	50	12	23.1
6. Power supply required for certain design	1	1.9	1	1.9	14	26.9	26	50	10	19.2
7. Spread of infectious disease from poor hygiene	3	5.8	4	7.7	12	23.1	18	34.6	15	28.8
8. Unknown safety concerns	3	5.8	1	1.9	9	17.3	27	51.9	12	23.1

### 5.4 Application of exoskeleton

In this section, the respondents were asked about their opinion on the kind of suitable tasks that could use exoskeletons. Of the 52 respondents, 8 (15.4%) suggested the welding area, while 14 respondents (26.9%) suggested a material handling area as it requires heavy loading daily. Meanwhile, 7 respondents (13.5%) added that the assembly line would be a suitable sector for exoskeleton as it includes repetitive and handling tasks. Two of the respondents (3.8%) also suggested exoskeleton could be applied in the whole production line. In general, 20 respondents (38.5%) suggested applying exoskeleton to assembly tasks, while 2 suggested tyre assembly, 3 chassis assembly, 2 engine and transmission assembly, 2 sub-assembly, and 4 underbody assembly. Table 5 illustrates the detailed results.

**Table 5:** Application of exoskeleton.

Recommended tasks		
	Frequency	Percentage (%)
Welding	8	15.4
Material handling	14	26.9
Painting process	2	3.8
Assembly	7	13.5
Tyre assembly	2	3.8
Chassis assembly	3	5.8
Engine & transmission assembly	2	3.8
Underbody assembly	4	7.7
Sub assembly	2	3.8
Repairing tasks	6	11.5
All sections	2	3.8

### 5.5 Design features of the exoskeleton

More than half of the respondents (n = 27, 51.9%) were opinionated that the repair and maintenance of the exoskeleton are extremely important. While 21 respondents (40.4%) were neutral regarding the overall appearance of the exoskeleton, where 4 of them rated that appearance is not important. The training duration for users to become proficient was rated very important by 32 respondents (61.5%). Respondents’ perspectives on the design features of the exoskeleton are summarised in Table 6.

**Table 6:** Design features of exoskeleton.

Design Features of exoskeleton	Not important at all		Not very important		Neither important nor not important		Very important		Extremely important	
	Freq.	%	Freq.	%	Freq.	%	Freq.	%	Freq.	%
1. Repair and maintenance	2	3.8	1	1.9	6	11.5	16	30.8	27	51.9
2. Ability to lift heavy load	0	0.0	1	1.9	7	13.5	21	40.4	23	44.2
3. Range of battery life	0	0.0	2	3.8	8	15.4	20	38.5	22	42.3
4. Overall appearance	4	7.7	4	7.7	21	40.4	14	26.9	9	17.3
5. Portability of the device	0	0.0	0	0.0	7	13.5	19	36.5	26	50.0
6. Length of training to become proficient	0	0.0	0	0.0	7	13.5	32	61.5	13	25.0
7. Ease of putting the suit on and taking it off	0	0.0	1	1.9	9	17.3	17	32.7	25	48.1
8. Purchase cost	1	1.9	0	0.0	2	3.8	16	30.8	33	63.5
9. Comfort	0	0.0	1	1.9	5	9.6	19	36.5	27	51.9
10. Weight of the exoskeleton suits	0	0.0	0	0.0	5	9.6	18	34.6	29	55.8

### 5.6 Opinion on exoskeleton

The final section of this survey required the respondents to provide their review on applying the exoskeleton technology into the industrial processes. Based on the observation, 8 respondents (15.4%) thought that it could support posture and ergonomics, while 9 (17.3%) indicated that it could minimise worker fatigue and work-related injuries. Approximately 25% (n = 13) of the respondents were excited about the advantages the new technology could bring to their workplace, whereas 7 respondents (13.5%) suggested that the exoskeleton can reduce energy usage by workers and maximise accomplished work. Meanwhile, for 4 of the respondents (7.7%) the exoskeleton technology resembled some comic or movie character. According to 5 respondents (9.6%), exoskeleton technology can assist workers to lift heavy items. Also, 4 respondents (7.7%) thought about the safety design and cost required when told about the exoskeleton technology, whereas 2 respondents (3.8%) viewed the exoskeleton as a type of robotic and were unsure how it could be used at a workplace.

In the next question, the respondents were asked about their expectations if exoskeletons were applied at their workplace. Based on their responses, 15 (28.8%) expected that the exoskeleton could increase the productivity of workers, while 14 (26.9%) claimed that workplace injuries could be reduced with the exoskeleton in place. Moreover, 4 respondents (7.7%) stated that it could help to reduce the need for manpower and increase their productivity, whereas 4 others (7.7%) presumed that it could increase workers’ efficiency while performing specific tasks. The exoskeleton is also expected to help workers to lift heavy parts or equipment easily (n = 7, 13.5%), increase the safety of the workplace (n = 4, 7.7%), and reduce the cost for workers and injury (n = 3, 5.8%).

A majority of the respondents (n = 45, 86.5%) suggested that the exoskeleton can be suitably applied in the existing workplace, while 7 respondents (13.5%) thought that the technology was not suitable. Of the total, 18 respondents (24.6%) rated scale 4 to state that the application of exoskeleton in the workplace is ‘very required’, while 11 (21.2%) respondents ranked that exoskeleton is most required (scale 5). However, 3 respondents (5.8%) claimed that the use of an exoskeleton is not necessary.

Regarding the suitability of the exoskeleton application, 25 respondents (48.1%) indicated that it is suitable to be applied. The 25 respondents claimed that the exoskeleton could help increase the efficiency of workers (n = 3, 5.8%), protect the workers from injury (n = 4, 7.7%), reduce manpower (n = 2, 3.8%), and help lift heavy items and equipment (n = 11, 21.2%). However, 2 (3.8%) of the respondents from the 25 thought it would be suitable if the limitations are reduced. On the other hand, 22 (42.3%) respondents indicated that the exoskeleton is not suitable to be applied in current production. The 22 respondents indicated that the exoskeleton is not suitable because the exoskeleton could make the tasks more difficult to perform (n = 3, 5.8%), the suit is costly (n = 10, 19.2%), it would require more skills and difficult to maintain (n = 2, 3.8%), the current tasks are not heavy (n = 3, 5.8%), the working environment is oily and could increase unknown risks (n = 1, 1.9%), it cannot be flexible for tasks that require flexibility (n = 1, 1.9%), and that the current process planning or equipment are already sufficient to complete the tasks (n = 2, 3.8%).

As for the disadvantages of the exoskeleton, 22 respondents (42.3%) highlighted that the cost of the exoskeleton could affect its acceptance and 6 others (11.5%) were concerned about the maintenance cost. The respondents also added that the size of the exoskeleton could affect its acceptance as the working space is limited (n = 6, 11.5%) and the exoskeleton lack flexibility, hence, might restrict workers' movement (n = 4, 7.7%). The ease of maintenance was highlighted by 2 of the respondents (3.8%), whereas reliability and durability of the exoskeleton were concerns of 3 respondents (5.8%). Also, 3 (5.8%) others mentioned the limitation of usage area and 1 (1.9%) respondent was concerned about the battery. Nevertheless, 3 other respondents (5.8%) had no idea as they have never used an exoskeleton.

Regarding the safety of the device, 29 respondents (55.8%) stated that equipment safety is extremely important, 16 (30.8%) thought that it is very important, while 7 (13.5%) were neutral. The respondents also shared their opinions on their concerns regarding the safety of the exoskeleton. Seven of the respondents (13.5%) were concerned about the hygiene if the same exoskeleton is shared, while 20 respondents (38.5%) were more concerned about the risk of injury due to the wrong operation of the suit. Meanwhile, 7 respondents (13.5%) mentioned that limited movement while wearing an exoskeleton could cause accidents and serious injury. Two of the respondents (3.8%) were concerned about the weight of the exoskeleton, while 2 (3.8%) others were worried about users falling while wearing an exoskeleton. Some of the respondents were also concerned about the unpredicted malfunction of the exoskeleton (n = 5, 9.6%), power supply (n = 3, 5.8%), and 4 others (7.7%) were clueless as they had no experience using an exoskeleton.

Of the 52 respondents, 18 (34.6%) thought that the workers in their industry are ready for the exoskeleton application, while 23 (44.2%) of them claimed that the workers are not ready for the exoskeleton. Five of the respondents (9.6%) were not very sure because they were not sure about the skills required, whereas, 4 respondents (7.7%) claimed that training is a must to operate the exoskeleton.

The respondents (n = 34, 65.4%) also suggested that if the workers are not ready for the exoskeleton, they might not consider the application of the exoskeleton suit at their workplace. The remaining 18 (34.6%) respondents indicated that the skills of workers would not affect their decision on the application of exoskeleton.

On the other hand, 37 respondents (71.2%) indicated that education and training is a must for the workers to operate an exoskeleton. Two of the respondents (3.8%) highlighted that the design must be user friendly. However, 5 respondents (9.6%) stated that education will not affect the usage of the exoskeleton. All the responses are tabulated in Table 7.

**Table 7:** Opinion of exoskeleton.

Opinions on exoskeleton		
	Frequency	Percentage (%)
The first perspective of the exoskeleton - Support for posture and ergonomics - Minimise work fatigue and work-related injuries - New technology and exciting - Reduce energy usage by workers and maximise work done - Movie or comic characters - Assist workers to lift heavy items - Safety design and high cost - No idea how robotics could help	8 9 13 7 4 5 4 2	15.4 17.3 25 13.5 7.7 9.6 7.7 3.8
Expected output of exoskeleton - Increase productivity - Reduce workplace injury - Reduce manpower - Increase efficiency of workers - Lift heavy parts and equipment - Increase safety - Reduce the cost of workers and cost of injuries	15 14 4 4 7 4 3	28.8 26.9 7.7 7.7 13.5 7.7 5.8
Acceptance of exoskeleton in the workplace - Yes - No	45 7	86.5 13.5
The need for exoskeleton in the workplace - Not necessary - Neither necessary nor not necessary - Necessary - Very require - Most required	3 4 16 18 11	5.8 7.7 30.8 34.6 21.2
Is the exoskeleton suitable for the workplace? **Suitable** - Increase efficient - Protect workers from injury - Reduce manpower - Easily lift heavy items and equipment - Existing limitations must be reduced **Not suitable** - If existing tasks become difficult with the use of exoskeleton - High cost - More skills and maintenance - Current tasks are not heavy - Environment not suitable - Tasks require workers to be flexible - Current process planning, equipment is enough to run production	3 4 2 11 2 3 10 2 3 1 1 2	5.8 7.7 3.8 21.2 3.8 5.8 19.2 3.8 5.8 1.9 1.9 3.8
Advantages of exoskeleton - Help lift equipment - Back support - Minimise of injury - Protect the workers - Support body movement - Reduce workload - Assist in work - Not attractive	9 7 7 6 7 1 3 3	17.3 13.5 13.5 11.5 13.5 1.9 5.8 5.8
Disadvantages of exoskeleton - Cost of exoskeleton - Maintenance cost - Size of exoskeleton - Lack of flexibility and restriction of movement - Ease of maintenance - Reliability and durability - Limitation of usage area - Battery duration - No idea without experience in using an exoskeleton	22 6 6 4 2 3 3 1 3	42.3 11.5 11.5 7.7 3.8 5.8 5.8 1.9 5.8
The importance of exoskeleton's safety - Neither important nor not important - Very important - Extremely important	7 16 29	13.5 30.8 55.8
Safety concern of exoskeleton - Hygiene - Risk of other injury - Limited movement - Weight of exoskeleton - Users falling when wearing an exoskeleton - Unpredicted malfunction - Power supply - No idea	7 20 7 2 2 5 3 4	13.5 38.5 13.5 3.8 3.8 9.6 5.8 7.7
Workers' readiness to use exoskeleton - Ready - Not ready - Not sure - Training is a must	18 23 5 4	34.6 44.2 9.6 7.7
The decision affected by workers' skills - Yes - No	34 18	65.4 34.6
Is training or education important for workers? - Education and training are a must - Design must be user friendly - Not important	37 2 5	71.2 3.8 9.6

## 6 Discussion

The participants in this study were generally positive about the exoskeleton technology. However, most of the respondents were concerned about the cost incurred for the implementation and maintenance of the exoskeleton technology. According to the results, most of the respondents recommended the application of exoskeleton in material handling tasks, followed by welding processes (especially in the bus industry), and different types of assembly processes (engine assembly etc.). The weight of the exoskeleton is also an important concern among the respondents. Besides the cost, some of the respondents indicated that the exoskeleton is not suitable to be applied in their industry because the existing process is already well designed and the tasks are not heavy because the exoskeleton might make the process more complicated. More than half of the respondents also claimed that the workers at their workplace might not be ready for the application of such technology, hence, they might not accept the usage of an exoskeleton, Hence, training and educating the existing workers is very important for any industry.

All the 52 respondents were separated into 3 groups; 31 from the passenger car assembly industry, 9 from truck assembly industry, and 12 from bus assembly industry. Items in the Likert-scale which scored more than 80% for scale option 4 and 5 were discussed in this section. According to the respondents from the passenger car assembly industry, the three main benefits of using an exoskeleton include reduced muscle fatigue, minimised WMSD, and increased maximum lifting. Their main concern on its limitation included the high purchase cost and limited movement. The recommended processes that are suitable for the application of exoskeleton include material handling, repairing, and assembly tasks. The respondents also expressed their concern on the design features particularly on the ability to lift a heavy load, purchase costing, and weight of the exoskeleton.

Employees in the truck industry were interested in the reduction in time due to injuries, increased work productivity, and reduced muscle fatigue. Although the flexibility of the exoskeleton is deemed as a limitation in this industry, the top-recommended tasks for the application of the exoskeleton include material handling, assembly, and chassis assembly tasks. Similarly, the employees of the truck industry were also concerned about the purchase cost, portability, and the weight of the exoskeleton.

Meanwhile, respondents from the bus industry were interested in the application of exoskeleton due to the reduction in time due to injuries, increased productivity, and minimised injuries cost. The biggest limitation was the restricted movement, flexibility, adjustability for different users, and unforeseen accidents or malfunctions that might cause safety concerns among workers. These employees suggested the welding, material handling, and assembly sectors for the application of the exoskeleton. The design features that were of concern among the bus industry employees included purchase cost, length of training, repair and maintenance of exoskeleton, apart from the comfort for users.

According to Deborah [7], previous studies revealed that the exoskeleton is accepted by users who understand the benefits of the device for their purposes. Contrarily, Shah [38] highlighted that the exoskeleton devices did not achieve their requirement in healthcare applications. According to Wolff's [39] study on the perspectives of healthcare professionals and potential users of exoskeleton technology, the factor which was considered most important was comfort and safety of exoskeleton. The respondents in the study were concerned of the potential harm and risk of falling, hence, added that wheelchairs would be a better option for now. According to Jung [40], clinicians were positive about the concept of exoskeleton technology, while the older adults commented that they might not require an exoskeleton. According to the study, exoskeleton design should focus on the ease of use to support the elderly (particularly to switch the device on and off) during their daily activities like walking, lifting, climbing and standing.

This survey involved employees from the automotive assembly industry voluntarily. In the survey, respondents were prompted to provide their views on exoskeleton technology supported by pictures and general description as an introduction for the exoskeleton devices. As some of the respondents could not fully understand the characteristics of the exoskeleton, their feedback was only based on the explanation provided in the questionnaire. However, with the continuous development of exoskeleton technology, the application of exoskeleton into the industry will become feasible in the future. More data and studies are required to design suitable features required in specific industries to support workers to conduct daily tasks and reduce the chances of injuries using the exoskeleton technology.

## 7 Application

The research would be very useful in the application of exoskeleton in the automotive assembly industry in Malaysia. Through the findings of survey, it shows the feedback on the application of exoskeleton by the practitioner which offered the insights of successful acceptance of exoskeleton application into the industry. The data is crucial for the exoskeleton developer to design exoskeleton that can fulfil the requirement of automotive assembly industry.

## 8 Conclusion

This online study successfully assessed the perspectives and acceptance of exoskeleton technology in the automotive industry. Most of the automotive employees accepted the application of the exoskeleton at their workplace, as they expected the exoskeleton to increase productivity and reduce workplace injury. Material handling was the most preferred task for the application of an exoskeleton. Most of the respondents also indicated that the overall appearance of the exoskeleton is not as important as its application. The costing and maintenance cost of the exoskeleton remains a concern among many employees. Nearly half of the respondents indicated that the workers in their workplace are not ready for the application of such technology, hence, training and education would be beneficial for them. To ensure the suitability of the exoskeleton devices in the Malaysian automotive assembly industry, additional research and development based on the employees’ suggestions are required. Different productions will require different tasks, hence, the exoskeletons would require different specifications to fit the industry needs. This where different types of industries recommend different application of exoskeleton varying with the tasks. The passenger car industry recommended the application into material handling, repairing and assembly line, truck industry recommended for material handling, assembly line and chassis assembly line while the bus industry prefers to apply into welding tasks, material handling tasks and assembly line. To avoid confusion, some detailed questions on the exoskeleton were not included in this questionnaire. Based on the number of employees among all the automotive assembly industries in Malaysia as shown in Table 1, the requirement for manpower is high as many of the processes is highly dependent on manual operations. Therefore, the application of exoskeleton is able to reduce the injuries and assist workers to conduct their task which means there is a high potential to apply the exoskeleton in the industry. With the funding for further research, both the exoskeleton makers and automotive assembly industry would be able to learn about the potential applications and requirements of exoskeleton technology in the industries. This would help in the development of exoskeleton to cater for the specific needs in the industry. In short, further studies into areas like the costing of the devices, comfortability, and portability could increase the acceptance level. To optimise industry utility and uptake, it is necessary to re-evaluate and improve based on industry opinions. The limitation of this research is the data collected was specifically from the automotive assembly industries in Malaysia and the findings are concerned about the local needs where the data gathered is through questionnaire.

## 9 Key points

• The results show most of the respondent are interested in the application of exoskeleton.
• Cost of implement exoskeleton and maintenance plays as very important role to most of the respondent.
• The requirement and comments from different industry highlighted the varying needs of the industries based on their process and production line. However, material handling is the most common area which seems to be the choice for the application of exoskeleton in the industry.

## Data availability statement

The data has not been deposited into a publicly available repository. Data will be made available on request.

## CRediT authorship contribution statement

**Woun Yoong Gan:** Writing – original draft, Data curation, Conceptualization. **Raja Ariffin Raja Ghazilla:** Writing – review & editing, Supervision, Methodology, Conceptualization. **Hwa Jen Yap:** Writing – review & editing, Supervision, Conceptualization. **Suman Selvarajoo:** Resources, Methodology.

## Declaration of competing interest

The authors declare that they have no known competing financial interests or personal relationships that could have appeared to influence the work reported in this paper.
